# Lessening Production

**Published:** 1874-11

**Authors:** 


### LESSENING PRODUCTION.

A PROMINENT manufacturer of cotton goods, whose factories are in Rhode Island, informed us a day or two since that parties engaged in his line of production had been doing business at a loss all summer, and that a movement was on foot to have the principal factories run on half time through the winter, the hope being that by thus lessening the stock of goods business might be afterwards done at a profit. Since the panic, a year ago, consumption has greatly diminished, economy having been almost universally practiced. While this course must result in general good, its effect upon manufacturers is, for the time, very disheartening. This condition can only be met by diminished production, to meet the lessened demand.

